# 4-Hy­droxy-5-meth­oxy-*N*,1-dimethyl-2-oxo-*N*-[4-(tri­fluoro­meth­yl)phen­yl]-1,2-di­hydro­quinoline-3-carboxamide

**DOI:** 10.1107/S1600536814003031

**Published:** 2014-02-15

**Authors:** Emmanuel S. Akinboye, Ray J. Butcher, Sema Ozturk Yildirim, John T. Isaacs

**Affiliations:** a The Sidney Kimmel Comprehensive Cancer Center at Johns Hopkins University, 1650 Orleans Street, Baltimore, MD 21287, USA; b Department of Chemistry, Howard University, 525 College Street NW, Washington DC 20059, USA; c Department of Physics, Faculty of Sciences, Erciyes University, 38039 Kayseri, Turkey

## Abstract

The title compound, C_20_H_17_F_3_N_2_O_4_, named tasquinimod, is a second-generation oral quinoline-3-carboxamide analogue, which is currently in phase III clinical trials for the treatment of metastatic prostate cancer. The quinoline unit is almost planar (r.m.s. deviation of fitted atoms = 0.0075 Å). The carboxamide side chain, substituted at position 3, is tilted by 88.07 (7)° to the quinoline plane. Both the methyl and carbonyl groups of this carboxamide side chain are in a *syn* conformation. The 4-(tri­fluoro­meth­yl)phenyl plane is inclined at 50.62 (17)° to the plane of the carboxamide side chain, and at 87.14 (4)° to the plane of the quinoline ring system. The 4-hy­droxy H atom acts as a double proton donor in an intra­molecular hydrogen bond to the 5-position meth­oxy O atom and in an inter­molecular contact to the 2-oxo group, generating a chain along [010] in the crystal structure.

## Related literature   

For background to the activity of the second generation quinolone-3-carboxamide analogues roquinimex (also known as linomide, systematic name: 4-hy­droxy-*N*,1-dimethyl-2-oxo-*N*-phenyl-1,2-di­hydro­quinoline-3-carboxamide) and tasquin­imod (systematic name: 4-hy­droxy-5-meth­oxy-*N*,1-dimethyl-2-oxo-*N*-[(4-tri­fluoro­meth­yl)phen­yl]-1,2-di­hydro­quinoline-3-carboxamide), see: Isaacs (2010 ▶). For similar structures, see: Dasari & Srikrishnan (2002 ▶); Jönsson *et al.* (2004 ▶); Jansson *et al.* (2006 ▶).
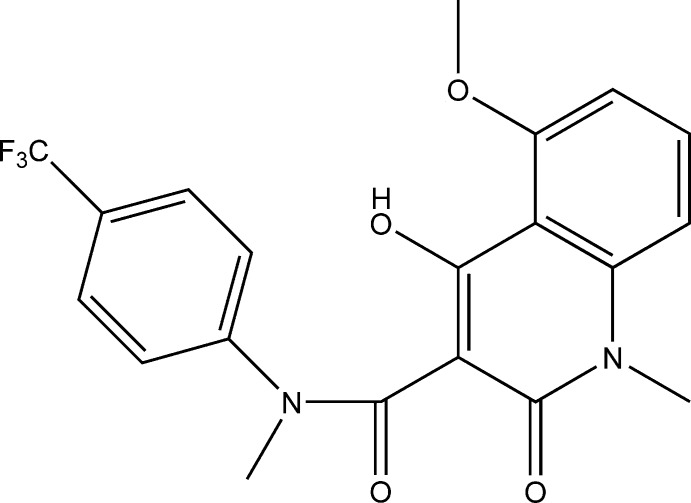


## Experimental   

### 

#### Crystal data  

C_20_H_17_F_3_N_2_O_4_
*M* *_r_* = 406.36
Monoclinic, 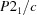

*a* = 10.8643 (9) Å
*b* = 10.6705 (9) Å
*c* = 15.8062 (13) Å
β = 105.696 (9)°
*V* = 1764.1 (3) Å^3^
*Z* = 4
Cu *K*α radiation
μ = 1.10 mm^−1^
*T* = 298 K
0.35 × 0.24 × 0.12 mm

#### Data collection  

Agilent Xcalibur (Ruby, Gemini) diffractometer
Absorption correction: multi-scan (*CrysAlis PRO*; Agilent, 2012 ▶) *T*_min_ = 0.741, *T*_max_ = 1.000
6559 measured reflections
3539 independent reflections
3010 reflections with *I* > 2σ(*I*)
*R*_int_ = 0.019

#### Refinement  

*R*[*F* ^2^ > 2σ(*F* ^2^)] = 0.044
*wR*(*F* ^2^) = 0.132
*S* = 1.06
3539 reflections
269 parameters
H atoms treated by a mixture of independent and constrained refinement
Δρ_max_ = 0.35 e Å^−3^
Δρ_min_ = −0.27 e Å^−3^

### 

Data collection: *CrysAlis PRO* (Agilent, 2012 ▶); cell refinement: *CrysAlis PRO*; data reduction: *CrysAlis PRO*; program(s) used to solve structure: *SHELXS97* (Sheldrick, 2008 ▶); program(s) used to refine structure: *SHELXL97* (Sheldrick, 2008 ▶); molecular graphics: *SHELXTL* (Sheldrick, 2008 ▶); software used to prepare material for publication: *SHELXTL*.

## Supplementary Material

Crystal structure: contains datablock(s) I, New_Global_Publ_Block. DOI: 10.1107/S1600536814003031/kp2464sup1.cif

Structure factors: contains datablock(s) I. DOI: 10.1107/S1600536814003031/kp2464Isup2.hkl

Supporting information file. DOI: 10.1107/S1600536814003031/kp2464Isup3.cml

CCDC reference: 986160

Additional supporting information:  crystallographic information; 3D view; checkCIF report

## Figures and Tables

**Table 1:** Hydrogen-bond geometry (Å, °)

*D*—H⋯*A*	*D*—H	H⋯*A*	*D*⋯*A*	*D*—H⋯*A*
O2—H2*O*⋯O4	0.89 (3)	1.82 (3)	2.5538 (16)	138 (2)
O2—H2*O*⋯O3^i^	0.89 (3)	2.28 (3)	2.8591 (17)	123 (2)

Symmetry code: (i) 
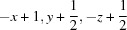
.

**Fig. 1.**
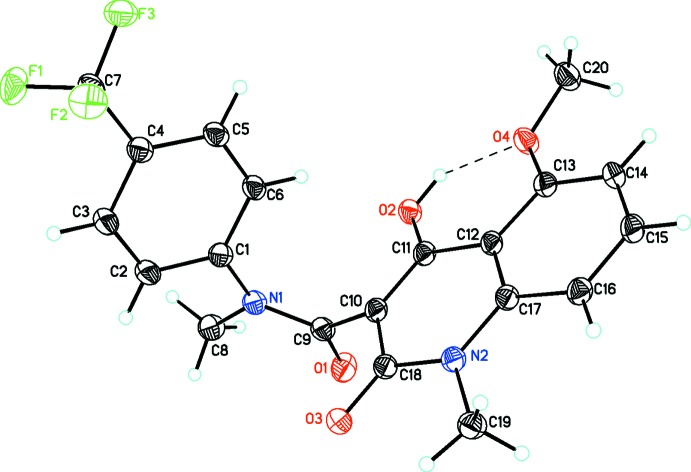
ORTEP diagram of the title compound showing the atom numbering and the atomic displacement parameters drawn at the 30% probability level. Intramolecular hydrogen bonding is shown by dashed lines.

**Fig. 2.**
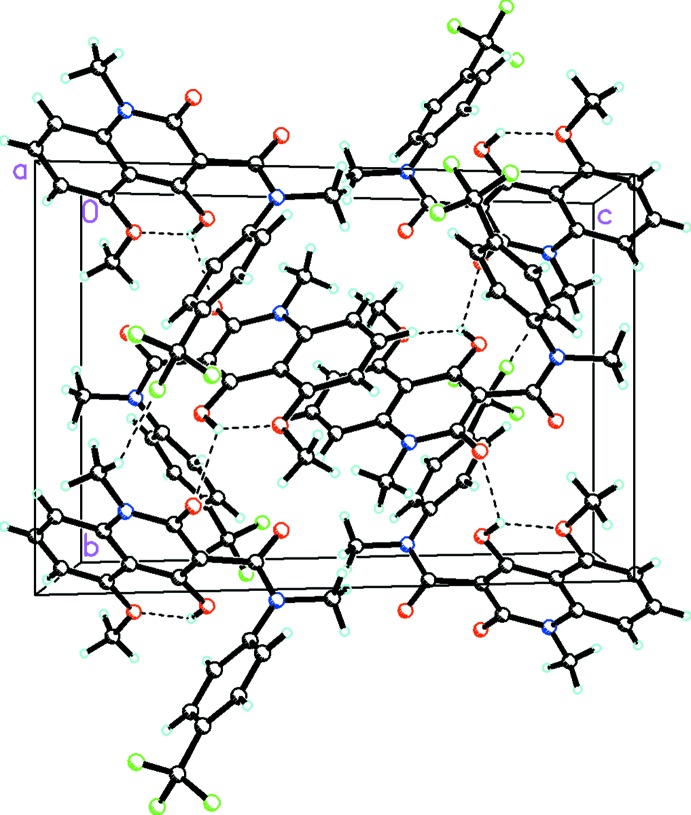
Crystal packing of the title compound viewed along the a axis. Hydrogen bonding is shown by dashed lines.

## supplementary crystallographic information

### 1. Comment

Roquinimex (also known as linomide: 4-hydroxy-N, 1-dimethyl-2-oxo-N phenyl-1,2-dihydroquinoline-3-carboxamide) is an orally active molecule that is therapeutic against solid malignancies *via* tumor selective anti-angiogenic abilities (Isaacs, 2010). In clinical trials, however, it had dose-limiting unacceptable side effects. From a library of second generation quinolone-3-carboxamide analogues, an analogue termed tasquinimod (*i.e.*, 4-hydroxy-5-methoxy-*N*,1-dimethyl-2-oxo-*N*-[(4-trifluoromethyl) phenyl]-1,2-dihydroquinoline-3-carboxamide) was identified being more than 30 times more potent than an anti-angiogenic agent without the toxic effects of roquinimex (Isaacs, 2010). Presently, tasquinimod is undergoing phase III clinical trials as monotherapy for castration resistant metastatic prostate cancer. Ultimately, tasquinimod will be optimally used in combination with other therapeutic agents. To identify the optimal combinational regime, the identification of the molecular target(s) involved in its mechanism of action is critical. Similar structures have been previously reported (Dasari *et al.*, 2002; Jönsson *et al.*, 2004; Jansson *et al.*, 2006).

As a lead for defining pharmacophoric features of tasquinimod, its three-dimensional structure was determined by X-ray structure analysis. The molecular structure shows that quinoline ring is almost planar (rms deviation of fitted atoms = 0.0075 Å). The carboxamide side chain at position 3, is tilted by 88.07 (7)° to the quinoline plane. Both, the methyl and carbonyl groups of this carboxamide side chain are in a *syn* conformation. The *N*-[4-trifluoromethyl)phenyl] plane is inclined at 50.62 (17)° to the plane of the carboxamide side chain, and 87.14 (4)° to the plane of the quinoline ring. The 4-hydroxy proton forms intramolecular hydrogen bond with the 5-position methoxy oxygen being planar to the quinoline ring (Fig. 1, Table 1). In the crystal packing molecules are linked by an intermolecular hydrogen bond between 4-hydroxy and 2-oxo groups generating a chain in the direction [010] (Fig. 2, Table 1).

### 2. Experimental

The preparation of the titled compound was reported by Jönsson *et al.* (2004).

To obtain crystal of this compound, a saturated solution of a pure sample of the title compound was made in 5 mL of acetonitrile (HPLC grade) at room temperature. The solution was filtered, and 2.5 mL of this saturated solution was transferred into a transparent 2 dram glass vial. Crystallization of this sample was by vapor diffusion in diethyl ether at room temperature over a period of 48 h. Crystal growth was observed within 24 h, but the experiment was left to stand for 48 h. A gentle vacuum filtration of the sample followed by air drying afforded white crystals of the compound for X-ray diffraction studies.

### 3. Refinement

H atoms were positioned geometrically and refined using the riding model, with C–H distance of 0.93–0.96 Å, with *U_iso_* (H) = 1.20 *U_eq_* (C) or 1.50 *U*_eq_ (C) for methyl H atoms. Hydrogen atoms involved in hydrogen bonding were refined isotropically.

### Crystal data

**Table:**

C_20_H_17_F_3_N_2_O_4_	*F*(000) = 840
*M**_r_* = 406.36	*D*_x_ = 1.530 Mg m^−^^3^
Monoclinic, *P*2_1_/*c*	Cu *K*α radiation, λ = 1.54184 Å
*a* = 10.8643 (9) Å	Cell parameters from 2882 reflections
*b* = 10.6705 (9) Å	θ = 2.9–75.7°
*c* = 15.8062 (13) Å	µ = 1.10 mm^−^^1^
β = 105.696 (9)°	*T* = 298 K
*V* = 1764.1 (3) Å^3^	Prism, colourless
*Z* = 4	0.35 × 0.24 × 0.12 mm

### Data collection

**Table:**

Agilent Xcalibur (Ruby, Gemini) diffractometer	3539 independent reflections
Radiation source: Enhance (Cu) X-ray Source	3010 reflections with *I* > 2σ(*I*)
Graphite monochromator	*R*_int_ = 0.019
Detector resolution: 10.5081 pixels mm^-1^	θ_max_ = 75.9°, θ_min_ = 4.2°
ω scans	*h* = −8→13
Absorption correction: multi-scan (*CrysAlis PRO*; Agilent, 2012)	*k* = −12→13
*T*_min_ = 0.741, *T*_max_ = 1.000	*l* = −19→19
6559 measured reflections	

### Refinement

**Table:**

Refinement on *F*^2^	Primary atom site location: structure-invariant direct methods
Least-squares matrix: full	Secondary atom site location: difference Fourier map
*R*[*F*^2^ > 2σ(*F*^2^)] = 0.044	Hydrogen site location: inferred from neighbouring sites
*wR*(*F*^2^) = 0.132	H atoms treated by a mixture of independent and constrained refinement
*S* = 1.06	*w* = 1/[σ^2^(*F*_o_^2^) + (0.079*P*)^2^ + 0.3326*P*] where *P* = (*F*_o_^2^ + 2*F*_c_^2^)/3
3539 reflections	(Δ/σ)_max_ < 0.001
269 parameters	Δρ_max_ = 0.35 e Å^−^^3^
0 restraints	Δρ_min_ = −0.27 e Å^−^^3^

### Special details

**Table:**

Geometry. All e.s.d.'s (except the e.s.d. in the dihedral angle between two l.s. planes) are estimated using the full covariance matrix. The cell e.s.d.'s are taken into account individually in the estimation of e.s.d.'s in distances, angles and torsion angles; correlations between e.s.d.'s in cell parameters are only used when they are defined by crystal symmetry. An approximate (isotropic) treatment of cell e.s.d.'s is used for estimating e.s.d.'s involving l.s. planes.
Refinement. Refinement of *F*^2^ against ALL reflections. The weighted *R*-factor *wR* and goodness of fit *S* are based on *F*^2^, conventional *R*-factors *R* are based on *F*, with *F* set to zero for negative *F*^2^. The threshold expression of *F*^2^ > σ(*F*^2^) is used only for calculating *R*-factors(gt) *etc*. and is not relevant to the choice of reflections for refinement. *R*-factors based on *F*^2^ are statistically about twice as large as those based on *F*, and *R*- factors based on ALL data will be even larger.

### Fractional atomic coordinates and isotropic or equivalent isotropic displacement parameters (Å^2^)

**Table:**

	*x*	*y*	*z*	*U*_iso_*/*U*_eq_	
F1	−0.01967 (12)	0.99424 (13)	0.21797 (7)	0.0584 (4)	
F2	0.02599 (11)	0.89869 (12)	0.34160 (8)	0.0513 (3)	
F3	0.15877 (10)	1.02840 (11)	0.31447 (8)	0.0480 (3)	
O1	0.42263 (14)	0.38668 (14)	0.11242 (8)	0.0479 (3)	
O2	0.59427 (11)	0.58973 (11)	0.24904 (7)	0.0341 (3)	
H2O	0.665 (2)	0.627 (3)	0.2800 (17)	0.056 (7)*	
O3	0.26656 (11)	0.31456 (11)	0.25976 (7)	0.0374 (3)	
O4	0.77617 (11)	0.61692 (12)	0.38907 (7)	0.0404 (3)	
N1	0.29733 (14)	0.55256 (14)	0.11795 (8)	0.0358 (3)	
N2	0.43399 (12)	0.33345 (13)	0.38337 (8)	0.0305 (3)	
C1	0.24402 (16)	0.64495 (15)	0.16254 (10)	0.0339 (3)	
C2	0.11175 (16)	0.66235 (17)	0.13832 (11)	0.0405 (4)	
H2A	0.0598	0.6100	0.0966	0.049*	
C3	0.05753 (16)	0.75701 (17)	0.17604 (11)	0.0404 (4)	
H3A	−0.0306	0.7682	0.1597	0.049*	
C4	0.13541 (15)	0.83560 (15)	0.23863 (10)	0.0338 (3)	
C5	0.26668 (15)	0.81696 (15)	0.26418 (10)	0.0334 (3)	
H5A	0.3184	0.8678	0.3072	0.040*	
C6	0.32089 (14)	0.72269 (15)	0.22568 (10)	0.0329 (3)	
H6A	0.4091	0.7115	0.2422	0.039*	
C7	0.07592 (15)	0.93888 (17)	0.27743 (10)	0.0365 (4)	
C8	0.25048 (19)	0.54745 (18)	0.02153 (10)	0.0442 (4)	
H8A	0.3207	0.5308	−0.0029	0.066*	
H8B	0.2122	0.6263	−0.0002	0.066*	
H8C	0.1880	0.4820	0.0048	0.066*	
C9	0.38280 (16)	0.46314 (16)	0.15651 (10)	0.0350 (3)	
C10	0.43151 (15)	0.45503 (15)	0.25502 (9)	0.0313 (3)	
C11	0.54421 (15)	0.50991 (15)	0.29655 (9)	0.0303 (3)	
C12	0.60720 (14)	0.47863 (15)	0.38701 (9)	0.0291 (3)	
C13	0.72526 (15)	0.53184 (16)	0.43456 (10)	0.0322 (3)	
C14	0.78219 (15)	0.49785 (16)	0.52027 (10)	0.0346 (3)	
H14A	0.8594	0.5336	0.5510	0.042*	
C15	0.72218 (16)	0.40882 (16)	0.56024 (10)	0.0362 (3)	
H15A	0.7602	0.3862	0.6183	0.043*	
C16	0.60834 (15)	0.35357 (15)	0.51625 (10)	0.0331 (3)	
H16A	0.5708	0.2938	0.5441	0.040*	
C17	0.54900 (14)	0.38831 (14)	0.42858 (9)	0.0297 (3)	
C18	0.36910 (14)	0.36439 (15)	0.29723 (10)	0.0313 (3)	
C19	0.37877 (16)	0.23443 (16)	0.42576 (10)	0.0361 (3)	
H19A	0.3656	0.2656	0.4796	0.054*	
H19B	0.4361	0.1642	0.4382	0.054*	
H19C	0.2984	0.2085	0.3873	0.054*	
C20	0.88302 (17)	0.6906 (2)	0.43465 (12)	0.0461 (4)	
H20A	0.9008	0.7526	0.3956	0.069*	
H20B	0.9563	0.6375	0.4554	0.069*	
H20C	0.8639	0.7314	0.4837	0.069*	

### Atomic displacement parameters (Å^2^)

**Table:**

	*U*^11^	*U*^22^	*U*^33^	*U*^12^	*U*^13^	*U*^23^
F1	0.0625 (7)	0.0633 (8)	0.0377 (5)	0.0276 (6)	−0.0067 (5)	−0.0050 (5)
F2	0.0575 (6)	0.0533 (7)	0.0479 (6)	−0.0070 (5)	0.0224 (5)	−0.0005 (5)
F3	0.0453 (5)	0.0401 (6)	0.0594 (6)	−0.0072 (5)	0.0155 (5)	−0.0122 (5)
O1	0.0600 (8)	0.0492 (8)	0.0284 (5)	0.0130 (6)	0.0014 (5)	−0.0064 (5)
O2	0.0374 (6)	0.0372 (6)	0.0250 (5)	−0.0019 (5)	0.0036 (4)	0.0034 (4)
O3	0.0362 (6)	0.0355 (6)	0.0356 (6)	−0.0014 (5)	0.0012 (4)	−0.0019 (5)
O4	0.0403 (6)	0.0441 (7)	0.0314 (5)	−0.0102 (5)	0.0005 (5)	0.0059 (5)
N1	0.0438 (7)	0.0350 (7)	0.0228 (6)	−0.0013 (6)	−0.0009 (5)	0.0007 (5)
N2	0.0337 (6)	0.0302 (6)	0.0261 (6)	0.0003 (5)	0.0056 (5)	0.0003 (5)
C1	0.0404 (8)	0.0312 (7)	0.0255 (7)	−0.0025 (6)	0.0010 (6)	0.0050 (6)
C2	0.0399 (8)	0.0364 (8)	0.0361 (8)	−0.0045 (7)	−0.0056 (6)	−0.0006 (6)
C3	0.0342 (7)	0.0389 (9)	0.0403 (8)	−0.0032 (7)	−0.0034 (6)	0.0011 (7)
C4	0.0369 (8)	0.0323 (8)	0.0285 (7)	−0.0033 (6)	0.0028 (6)	0.0038 (6)
C5	0.0368 (7)	0.0328 (8)	0.0261 (7)	−0.0057 (6)	0.0011 (6)	0.0029 (6)
C6	0.0330 (7)	0.0339 (8)	0.0273 (7)	−0.0029 (6)	0.0005 (5)	0.0041 (6)
C7	0.0340 (7)	0.0415 (9)	0.0298 (7)	−0.0032 (6)	0.0013 (6)	0.0030 (6)
C8	0.0584 (10)	0.0421 (9)	0.0245 (7)	−0.0007 (8)	−0.0020 (7)	0.0010 (6)
C9	0.0402 (8)	0.0346 (8)	0.0260 (7)	−0.0023 (6)	0.0017 (6)	−0.0022 (6)
C10	0.0363 (7)	0.0301 (7)	0.0240 (7)	0.0034 (6)	0.0020 (5)	−0.0003 (5)
C11	0.0353 (7)	0.0303 (7)	0.0237 (6)	0.0037 (6)	0.0054 (5)	0.0008 (5)
C12	0.0325 (7)	0.0297 (7)	0.0226 (6)	0.0040 (6)	0.0030 (5)	−0.0015 (5)
C13	0.0341 (7)	0.0320 (8)	0.0284 (7)	0.0019 (6)	0.0050 (6)	0.0004 (6)
C14	0.0326 (7)	0.0373 (8)	0.0286 (7)	0.0001 (6)	−0.0010 (6)	−0.0008 (6)
C15	0.0421 (8)	0.0380 (8)	0.0243 (7)	0.0035 (7)	0.0018 (6)	0.0026 (6)
C16	0.0397 (8)	0.0322 (7)	0.0260 (7)	0.0027 (6)	0.0068 (6)	0.0021 (6)
C17	0.0331 (7)	0.0302 (7)	0.0243 (6)	0.0035 (6)	0.0053 (5)	−0.0022 (5)
C18	0.0337 (7)	0.0299 (7)	0.0274 (7)	0.0030 (6)	0.0034 (6)	−0.0034 (6)
C19	0.0399 (8)	0.0349 (8)	0.0324 (7)	−0.0041 (6)	0.0080 (6)	0.0011 (6)
C20	0.0399 (8)	0.0497 (11)	0.0430 (9)	−0.0116 (8)	0.0013 (7)	0.0059 (8)

### Geometric parameters (Å, º)

**Table:**

F1—C7	1.3351 (19)	C5—H5A	0.9300
F2—C7	1.343 (2)	C6—H6A	0.9300
F3—C7	1.334 (2)	C8—H8A	0.9600
O1—C9	1.225 (2)	C8—H8B	0.9600
O2—C11	1.3440 (19)	C8—H8C	0.9600
O2—H2O	0.89 (3)	C9—C10	1.505 (2)
O3—C18	1.232 (2)	C10—C11	1.356 (2)
O4—C13	1.365 (2)	C10—C18	1.444 (2)
O4—C20	1.426 (2)	C11—C12	1.447 (2)
N1—C9	1.356 (2)	C12—C17	1.409 (2)
N1—C1	1.423 (2)	C12—C13	1.418 (2)
N1—C8	1.4713 (19)	C13—C14	1.377 (2)
N2—C17	1.390 (2)	C14—C15	1.396 (2)
N2—C18	1.3930 (19)	C14—H14A	0.9300
N2—C19	1.464 (2)	C15—C16	1.377 (2)
C1—C6	1.390 (2)	C15—H15A	0.9300
C1—C2	1.396 (2)	C16—C17	1.411 (2)
C2—C3	1.383 (3)	C16—H16A	0.9300
C2—H2A	0.9300	C19—H19A	0.9600
C3—C4	1.395 (2)	C19—H19B	0.9600
C3—H3A	0.9300	C19—H19C	0.9600
C4—C5	1.387 (2)	C20—H20A	0.9600
C4—C7	1.491 (2)	C20—H20B	0.9600
C5—C6	1.387 (2)	C20—H20C	0.9600
			
C11—O2—H2O	113.5 (17)	N1—C9—C10	120.79 (14)
C13—O4—C20	119.44 (13)	C11—C10—C18	122.75 (13)
C9—N1—C1	125.87 (12)	C11—C10—C9	119.65 (14)
C9—N1—C8	116.27 (14)	C18—C10—C9	116.03 (13)
C1—N1—C8	117.75 (14)	O2—C11—C10	116.88 (13)
C17—N2—C18	123.35 (13)	O2—C11—C12	122.92 (14)
C17—N2—C19	119.43 (12)	C10—C11—C12	120.16 (14)
C18—N2—C19	117.18 (13)	C17—C12—C13	118.84 (13)
C6—C1—C2	119.45 (16)	C17—C12—C11	117.81 (14)
C6—C1—N1	121.55 (15)	C13—C12—C11	123.33 (15)
C2—C1—N1	118.89 (14)	O4—C13—C14	123.74 (14)
C3—C2—C1	120.39 (15)	O4—C13—C12	115.12 (13)
C3—C2—H2A	119.8	C14—C13—C12	121.14 (15)
C1—C2—H2A	119.8	C13—C14—C15	118.94 (14)
C2—C3—C4	119.84 (15)	C13—C14—H14A	120.5
C2—C3—H3A	120.1	C15—C14—H14A	120.5
C4—C3—H3A	120.1	C16—C15—C14	121.90 (14)
C5—C4—C3	119.93 (16)	C16—C15—H15A	119.1
C5—C4—C7	120.80 (14)	C14—C15—H15A	119.1
C3—C4—C7	119.27 (15)	C15—C16—C17	119.54 (15)
C6—C5—C4	120.09 (14)	C15—C16—H16A	120.2
C6—C5—H5A	120.0	C17—C16—H16A	120.2
C4—C5—H5A	120.0	N2—C17—C12	120.19 (13)
C5—C6—C1	120.28 (15)	N2—C17—C16	120.19 (14)
C5—C6—H6A	119.9	C12—C17—C16	119.63 (14)
C1—C6—H6A	119.9	O3—C18—N2	121.36 (15)
F3—C7—F1	107.22 (15)	O3—C18—C10	122.93 (14)
F3—C7—F2	105.04 (13)	N2—C18—C10	115.69 (13)
F1—C7—F2	106.06 (14)	N2—C19—H19A	109.5
F3—C7—C4	113.18 (14)	N2—C19—H19B	109.5
F1—C7—C4	112.25 (13)	H19A—C19—H19B	109.5
F2—C7—C4	112.52 (15)	N2—C19—H19C	109.5
N1—C8—H8A	109.5	H19A—C19—H19C	109.5
N1—C8—H8B	109.5	H19B—C19—H19C	109.5
H8A—C8—H8B	109.5	O4—C20—H20A	109.5
N1—C8—H8C	109.5	O4—C20—H20B	109.5
H8A—C8—H8C	109.5	H20A—C20—H20B	109.5
H8B—C8—H8C	109.5	O4—C20—H20C	109.5
O1—C9—N1	121.14 (14)	H20A—C20—H20C	109.5
O1—C9—C10	118.07 (15)	H20B—C20—H20C	109.5
			
C9—N1—C1—C6	53.1 (2)	O2—C11—C12—C17	−176.45 (13)
C8—N1—C1—C6	−131.00 (17)	C10—C11—C12—C17	1.4 (2)
C9—N1—C1—C2	−130.73 (18)	O2—C11—C12—C13	1.9 (2)
C8—N1—C1—C2	45.2 (2)	C10—C11—C12—C13	179.78 (14)
C6—C1—C2—C3	0.7 (3)	C20—O4—C13—C14	−10.4 (2)
N1—C1—C2—C3	−175.56 (15)	C20—O4—C13—C12	169.82 (15)
C1—C2—C3—C4	0.0 (3)	C17—C12—C13—O4	178.95 (13)
C2—C3—C4—C5	−1.3 (3)	C11—C12—C13—O4	0.6 (2)
C2—C3—C4—C7	178.77 (15)	C17—C12—C13—C14	−0.9 (2)
C3—C4—C5—C6	1.9 (2)	C11—C12—C13—C14	−179.19 (15)
C7—C4—C5—C6	−178.19 (14)	O4—C13—C14—C15	−179.40 (15)
C4—C5—C6—C1	−1.2 (2)	C12—C13—C14—C15	0.4 (2)
C2—C1—C6—C5	−0.1 (2)	C13—C14—C15—C16	0.4 (3)
N1—C1—C6—C5	176.04 (14)	C14—C15—C16—C17	−0.8 (3)
C5—C4—C7—F3	19.4 (2)	C18—N2—C17—C12	−1.2 (2)
C3—C4—C7—F3	−160.76 (15)	C19—N2—C17—C12	176.37 (13)
C5—C4—C7—F1	140.90 (16)	C18—N2—C17—C16	179.06 (14)
C3—C4—C7—F1	−39.2 (2)	C19—N2—C17—C16	−3.4 (2)
C5—C4—C7—F2	−99.54 (18)	C13—C12—C17—N2	−179.28 (13)
C3—C4—C7—F2	80.34 (18)	C11—C12—C17—N2	−0.8 (2)
C1—N1—C9—O1	179.39 (16)	C13—C12—C17—C16	0.5 (2)
C8—N1—C9—O1	3.4 (2)	C11—C12—C17—C16	178.94 (14)
C1—N1—C9—C10	−0.6 (2)	C15—C16—C17—N2	−179.92 (14)
C8—N1—C9—C10	−176.58 (15)	C15—C16—C17—C12	0.3 (2)
O1—C9—C10—C11	83.9 (2)	C17—N2—C18—O3	−179.18 (14)
N1—C9—C10—C11	−96.11 (19)	C19—N2—C18—O3	3.2 (2)
O1—C9—C10—C18	−82.2 (2)	C17—N2—C18—C10	2.4 (2)
N1—C9—C10—C18	97.77 (18)	C19—N2—C18—C10	−175.13 (13)
C18—C10—C11—O2	177.94 (13)	C11—C10—C18—O3	179.83 (15)
C9—C10—C11—O2	12.8 (2)	C9—C10—C18—O3	−14.5 (2)
C18—C10—C11—C12	−0.1 (2)	C11—C10—C18—N2	−1.8 (2)
C9—C10—C11—C12	−165.21 (14)	C9—C10—C18—N2	163.83 (13)

### Hydrogen-bond geometry (Å, º)

**Table:**

*D*—H···*A*	*D*—H	H···*A*	*D*···*A*	*D*—H···*A*
O2—H2*O*···O4	0.89 (3)	1.82 (3)	2.5538 (16)	138 (2)
O2—H2*O*···O3^i^	0.89 (3)	2.28 (3)	2.8591 (17)	123 (2)

Symmetry code: (i) −*x*+1, *y*+1/2, −*z*+1/2.
